# Supplementary material for: AKT Mediates Adiponectin-Dependent Regulation of VSMC Phenotype
Source: Cells. 2023 Oct 20;12(20):2493. doi: 10.3390/cells12202493 (PMC10605922; doi:10.3390/cells12202493)

Supplemental Materials, Methods, and Figures

Methods

1.2 Genotyping

Mice were gently sedated with isoflurane to have tail tip collected and ears punched for identification. DNA from tails was isolated using the Quanta Accustart II Mouse Genotyping Kit (Quanta Biosciences, 95091). PCR was then run in a Mastercycler Nexus Gradient (Eppendorf). Primers sequences were obtained from Jackson Laboratory genotyping protocols database and were purchased from Sigma Aldrich. The sequence of the primers is shown in Table S1.

Table S1. Sequence of genotyping primers

Gene	**Primer ID**	**Sequence**	**Primer type**
ApoE	olMR0180	GCC TAG CCG AGG GAG AGC CG	Common Forward
	olMR0181	TGT GAC TTG GGA GCT CTG CAG C	WT reverse
	olMR0182	GCC GCC CCG ACT GCA TCT	Mutant reverse
Adipoq	28949	GGT GGC TCA CAA CCA TTC A	Common Forward
	28950	CTC CCA GGA GGT CTT CAT CA	WT reverse
	24955	CTT CCT GAC TAG GGG AGG AG	Mutant reverse

Table S2. List of antibodies

**Target antigen**	**Vendor or Source**	**Catalog #**	**Working concentration**	**Persistent ID / URL**
Rabbit phospho-Acetyl CoA Carboxylase (p-ACC, Ser79)	Cell Signaling	Cat#3676T	1:500	
Rabbit monoclonal Akt (Pan)	Cell Signaling	Cat#4691	1:1000	RRID:AB_915783
Rabbit monoclonal phospho-Akt (Ser 473)	Cell Signaling	Cat#4060	1:500	RRID:AB_2315049
Rabbit AMPK	Cell Signaling	Cat#5831S	1:500	RRID:AB_10622186
Rabbit p-AMPK (Thr172)	Cell Signaling	Cat#2535S	1:500	
Rabbit Cyclin D1	Cell Signaling	Cat#2922S	1:500	RRID:AB_2228523
Rabbit Decorin	Abcam	Cat#ab175404	1:1000	RRID:AB_2890261
Rabbit EGFR	Santa Cruz	Cat#sc-373746	1:500	RRID:AB_10920395
Rabbit Elastin	Abcam	Cat#ab217356	1:500	RRID:AB_2827685
Rabbit polyclonal ERK1/2	Cell Signaling	Cat#9102	1:1000	RRID:AB_330744

Rabbit monoclonal phospho-ERK1/2 (Thr202/Tyr 204)	Cell Signaling	Cat#4370	1:500	RRID:AB_2315112
Rabbit KLF4	Abcam	Cat#ab106629	1:1000	
Rabbit p-p38MAPK phosphor (Thr180/Tyr182)	Cell Signaling	Cat#9211	1:500	RRID:AB_331641
Rabbit p38	Cell Signaling	Cat#9212	1:1000	RRID:AB_330713
Rabbit MEK1/2	Cell Signaling	Cat#8727S	1:500	RRID:AB_10829473
Rabbit p-MEK (Ser 217/221)	Cell Signaling	Cat#9154S	1:500	RRID:AB_2138017
Rabbit myosin heavy chain (MHC)	Abcam	Cat#ab124205	1:500	RRID:AB_10974234
Rabbit myosin light chain (MLC)	Cell Signaling	Cat#3672S	1:500	RRID:AB_10692513
Rabbit p-MLC (Ser19)	Cell Signaling	Cat#3671S	1:500	RRID:AB_330248
Mouse myocardin	Sigma	Cat#SAB4200539	1:1000	
Rabbit monoclonal MMP2	Abcam	Cat#ab92536	1:1000	RRID:AB_10561597
Rabbit monoclonal MMP9	Abcam	Cat#ab76003	1:500	RRID:AB_1310463
Rabbit monoclonal MMP3	Abcam	Cat#ab52915	1:500	RRID:AB_881243
Rabbit Smad2/3 (.	Cell Signaling	Cat#8685T	1:500	RRID:AB_10889933
Rabbit SRF	Abcam	Cat#ab124205	1:1000	
Rabbit TAGLN/SM22	Abcam	Cat#ab14106	1:6000	RRID:AB_443021
Mouse monoclonal ACTA2/SM-Actin	Sigma-Aldrich	Cat#ABT1485	1:10000	
Mouse monoclonal TUBG1/Tubulin	Sigma-Aldrich	Cat#T5326	1:2000	RRID:AB_532292
Rabbit GADPH	Thermo Fisher Scientific	Cat#PA1-988	1:4000	RRID:AB_2107310
Rabbit p-mTOR (Ser2448)	Cell signaling	Cat#2971S	1:1000	RRID:AB_330970
Rabbit mTOR	Cell signaling	Cat# 2972S	1:1000	RRID:AB_330978
Rabbit p-p70S6K Thr389	Cell signaling	Cat#9205	1:2000	RRID:AB_330944

Table S3. Reagents

Description	*Source / Repository*	*Persistent ID / URL*
AdipoRon	Sigma Aldrich	Cat# SML0998-5MG
TCN (Triciribine, Akt inhibitor V)	Millipore	Cat#124012-1MG

2′,7′-dichlorodihydrofluorescein diacetate (H_2DCFDA)	Invitrogen	Cat#C6827
DHE	Life Technologies	Cat#D11347
MitoSox	Invitrogen	Cat#M36008
Erlotinib	Sigma Aldrich	Cat#SML2156
EZ Link Sulfo-NHS-Biotin	Pierce	Cat#21217
Metformin Hydrochloride	Sigma Aldrich	Cat#PHR1084-500MG
Immobilized Neutravidin	Pierce	Cat#29200
PD-98059 MEK inhibitor	Enzo Life Sciences	Cat#BML-E1360-0005
SB-525334 TGF-β inhibitor	VWR	Cat#103539118
Silver Stain Plus kit	Biorad	Cat#161-0449
ImageJ 6.0	Schneider et al., 2012	https://imagej.nih.gov/ij/
xPONENT		https://www.luminexcorp.com/xponent/
IBM (International Business Machines) SPSS 25 (Statistical Package for the Social Sciences version 25)		https://www.ibm.com/docs/en/spss-statistics/25.0.0

Supplemental Figures

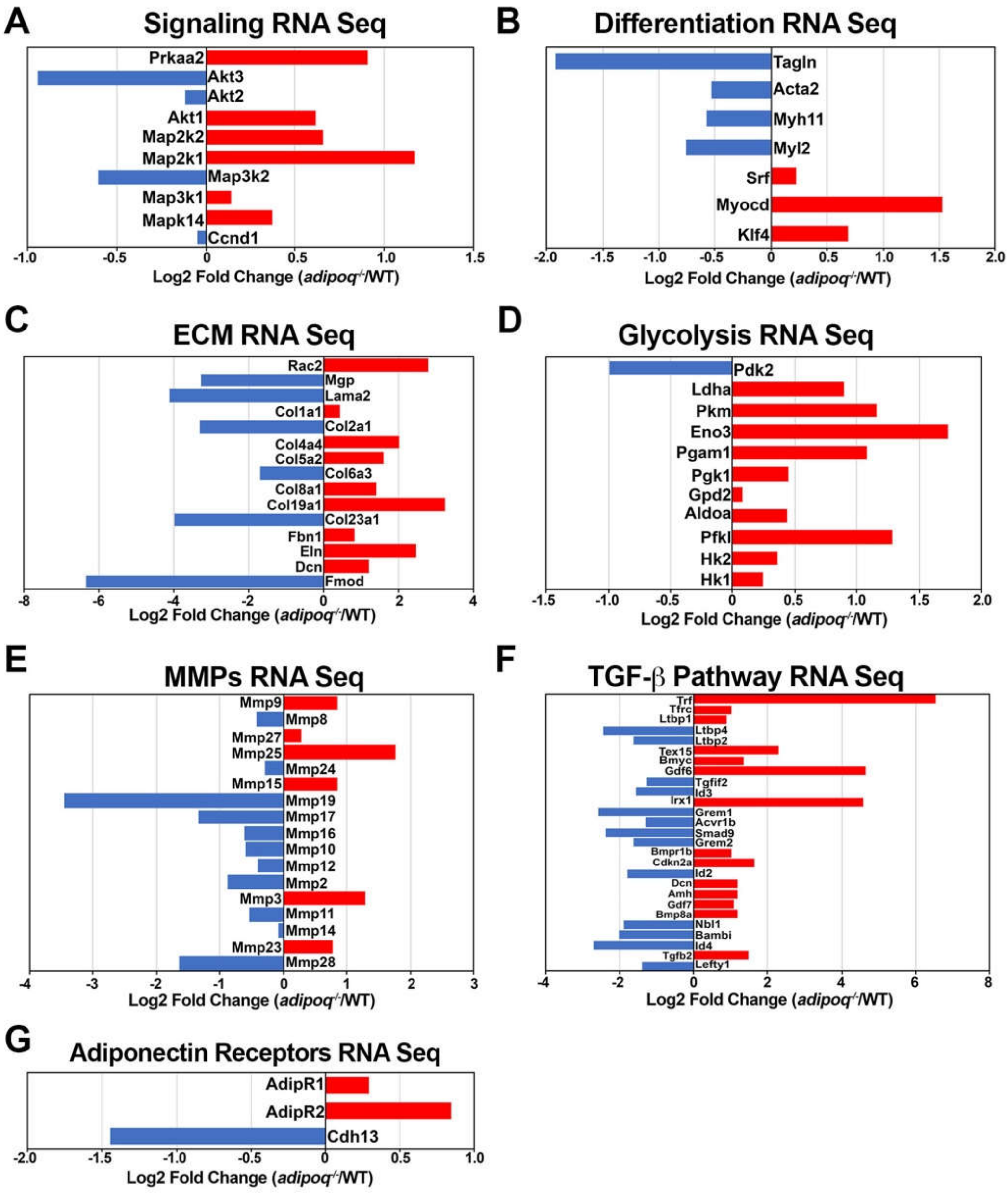

Figure S1. RNA expression profile of adiponectin deficient VSMCs. Changes in RNA expression of adipoq$^{-/-}$ compared with WT cells (log2 (fold change) is shown signaling (A), differentiation (B), ECM (C), glycolysis (D), MMPs (E), TGF-β (F) pathways and adiponectin receptors.

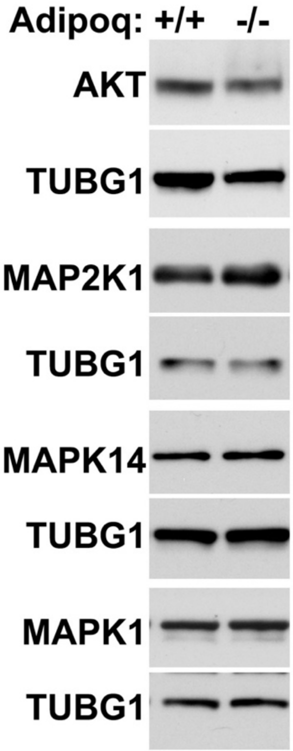

Figure S2. Protein expression of MAPK and AKT kinases in adiponectin deficient VSMCs. WT and adipoq$^{-/-}$ cells were separated in 4-20% precast gels and tested with the indicated antibodies. Quantification of the protein expression, adjusted by the loading control, is shown in Figure 2E.

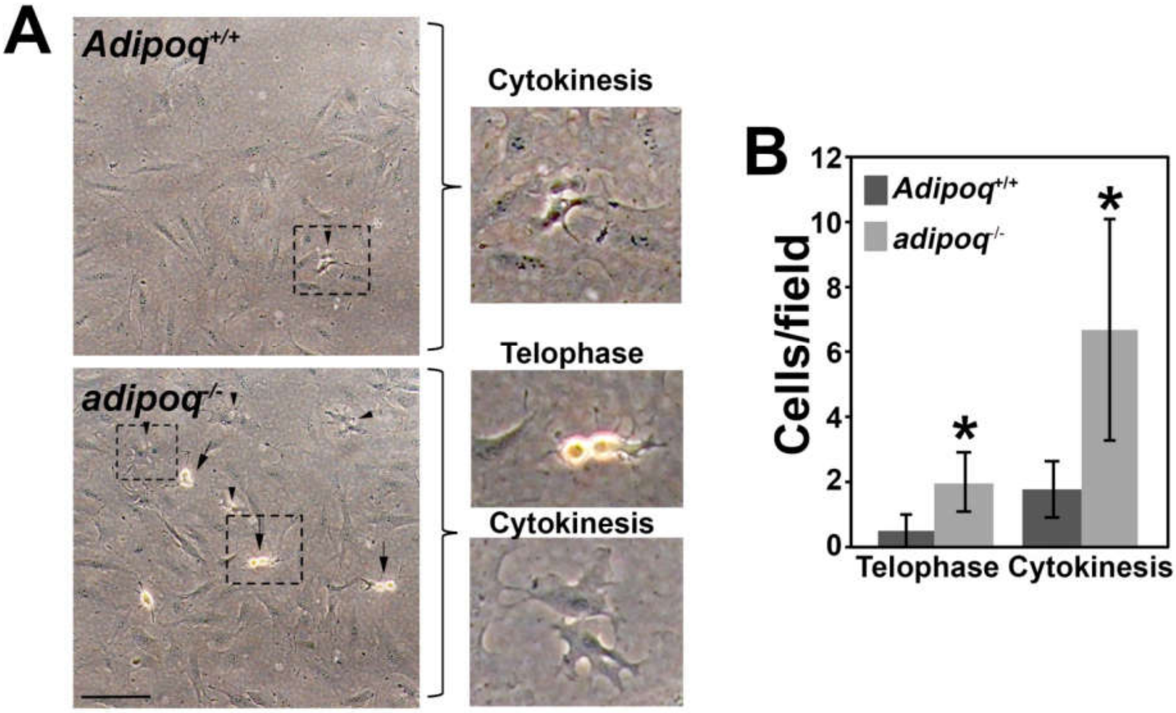

Figure S3. Adiponectin deficiency promotes proliferation. Cells cultured in media with10% FBS at 70-80% confluency were fixed in 0.2% glutaraldehyde and imaged in an Axio Observer A1 microscope (Carl Zeiss) with a 10x objective (scale bar = 100 mm) (A). Cells going through telophase and cytokinesis were quantified in 8 field/genotype (B). Asterisk (*) denotes $p<0.05$ compared with wild type cells.

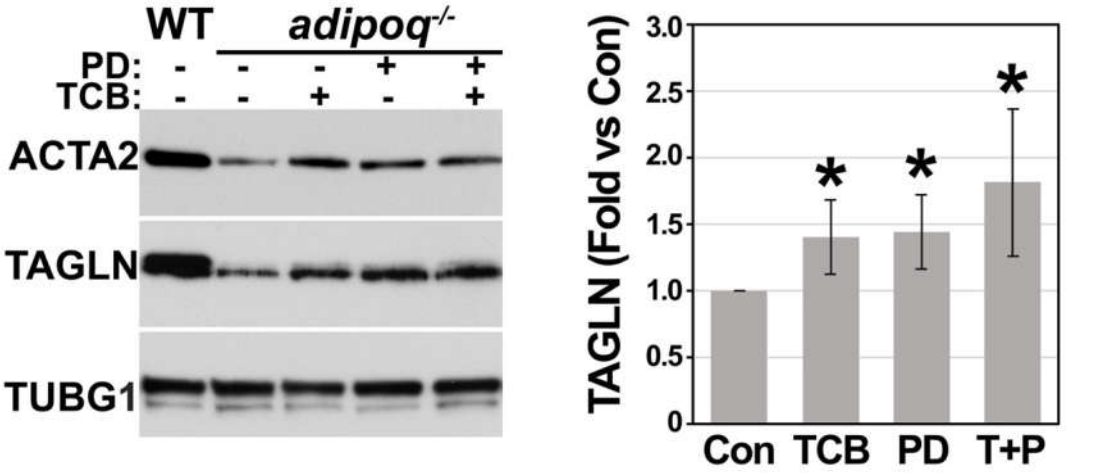

Figure S4. AKT and MAPK pathways regulates VSMC differentiation. Adiponectin deficient cells were starved in 0.2% FBS media for 24 h and then treated with 10 μM TCB, PD-98059 or both for 24 h. The expression of ACTA2 and TAGLN were quantified as fold compared with control adipoq$^{-/-}$ cells. Asterisk (*) denotes $p<0.05$. Wild type (WT) cells are shown in lane 1.

Original data for western blots

Figure 2C

Order antibodies:

1) pAKT, rabbit

stripping

2) pMEK, rabbit

stripping

3) p-p38, rabbit

4) pERK, rabbit

5) Tubulin, mouse

1) Cyclin D1, rabbit

2) Tubulin, mouse

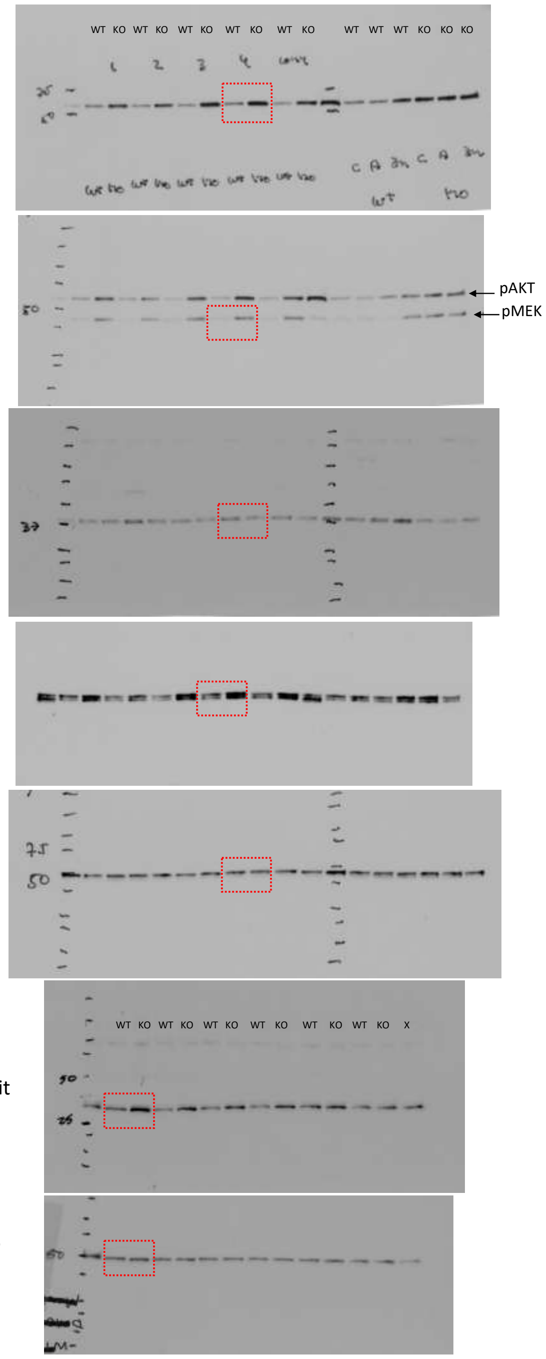

Figure 2C

1) Total AMPK, rabbit

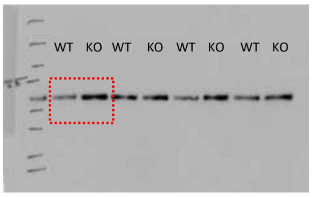

2) Tubulin, mouse

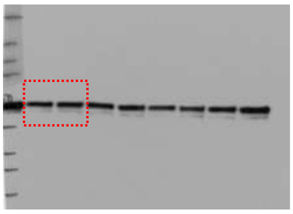

1) pAMPK, rabbit

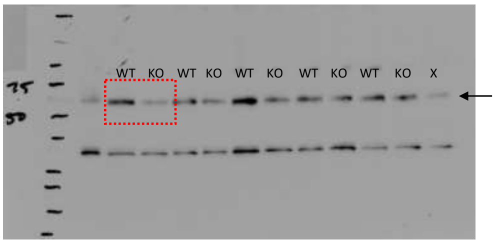

2) Tubulin, mouse

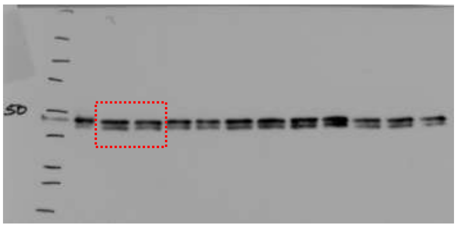

Figure 2D

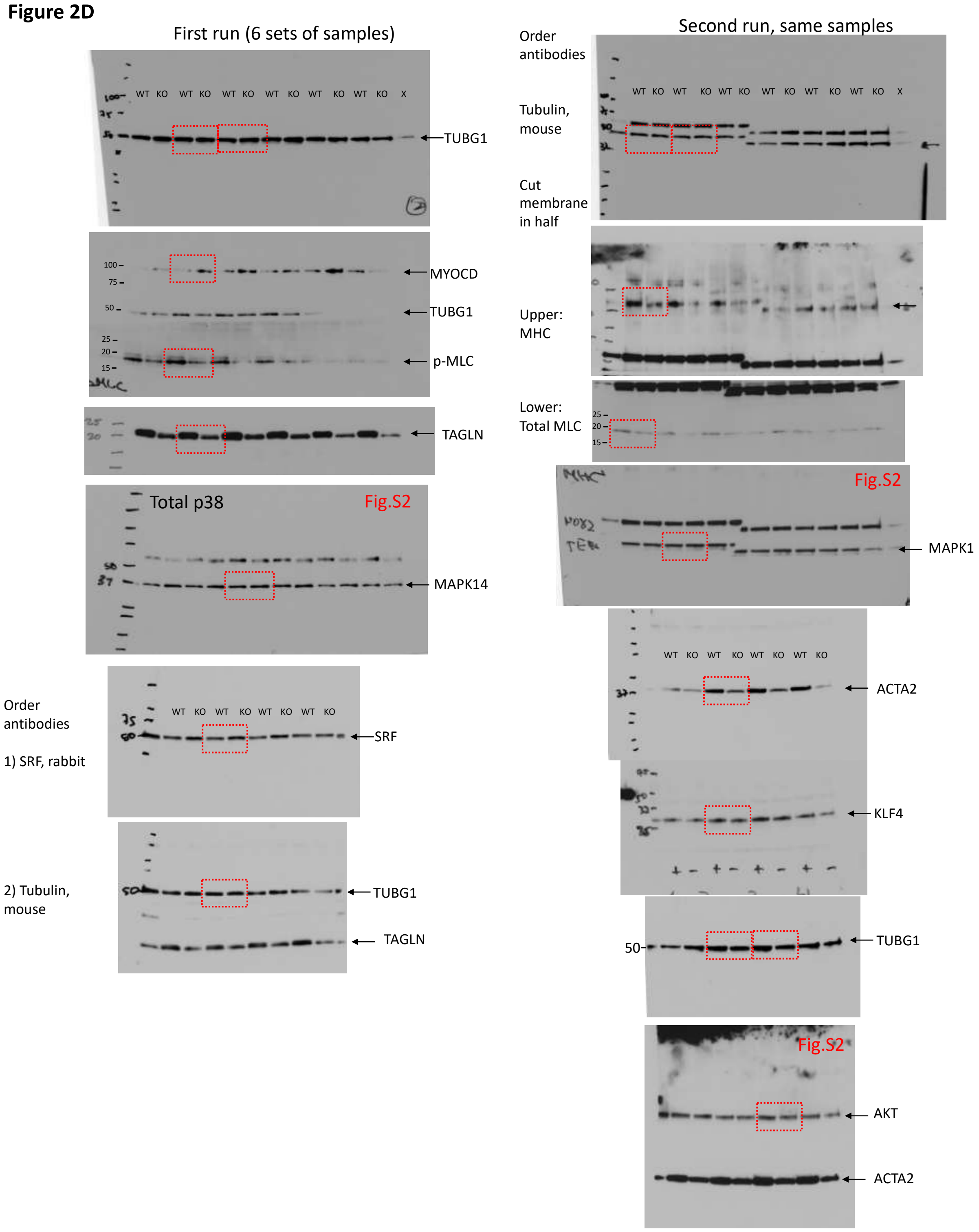
First run (6 sets of samples)
WT KO WT KO WT KO WT KO WT KO WT KO X
TUBG1
MYOCD
TUBG1
p-MLC
TAGLN
Total p38
Fig.S2
MAPK14
Order antibodies
1) SRF, rabbit
WT KO WT KO WT KO WT KO
SRF
2) Tubulin, mouse
TUBG1
TAGLN
Second run, same samples
Order antibodies
Tubulin, mouse
Cut membrane in half
WT KO WT KO WT KO WT KO WT KO WT KO X
Upper: MHC
Lower: Total MLC
Fig.S2
MAPK1
WT KO WT KO WT KO WT KO
ACTA2
KLF4
TUBG1
Fig.S2
AKT
ACTA2

Figure 2G

Order of antibodies for western blot:

1) KLF4, rabbit

Stripping

2) Myocardin, rabbit

Stripping

3) SRF, rabbit

SRF, low exposure

Cut membrane

4) Upper for Clathrin, mouse

Lower for Histone H3

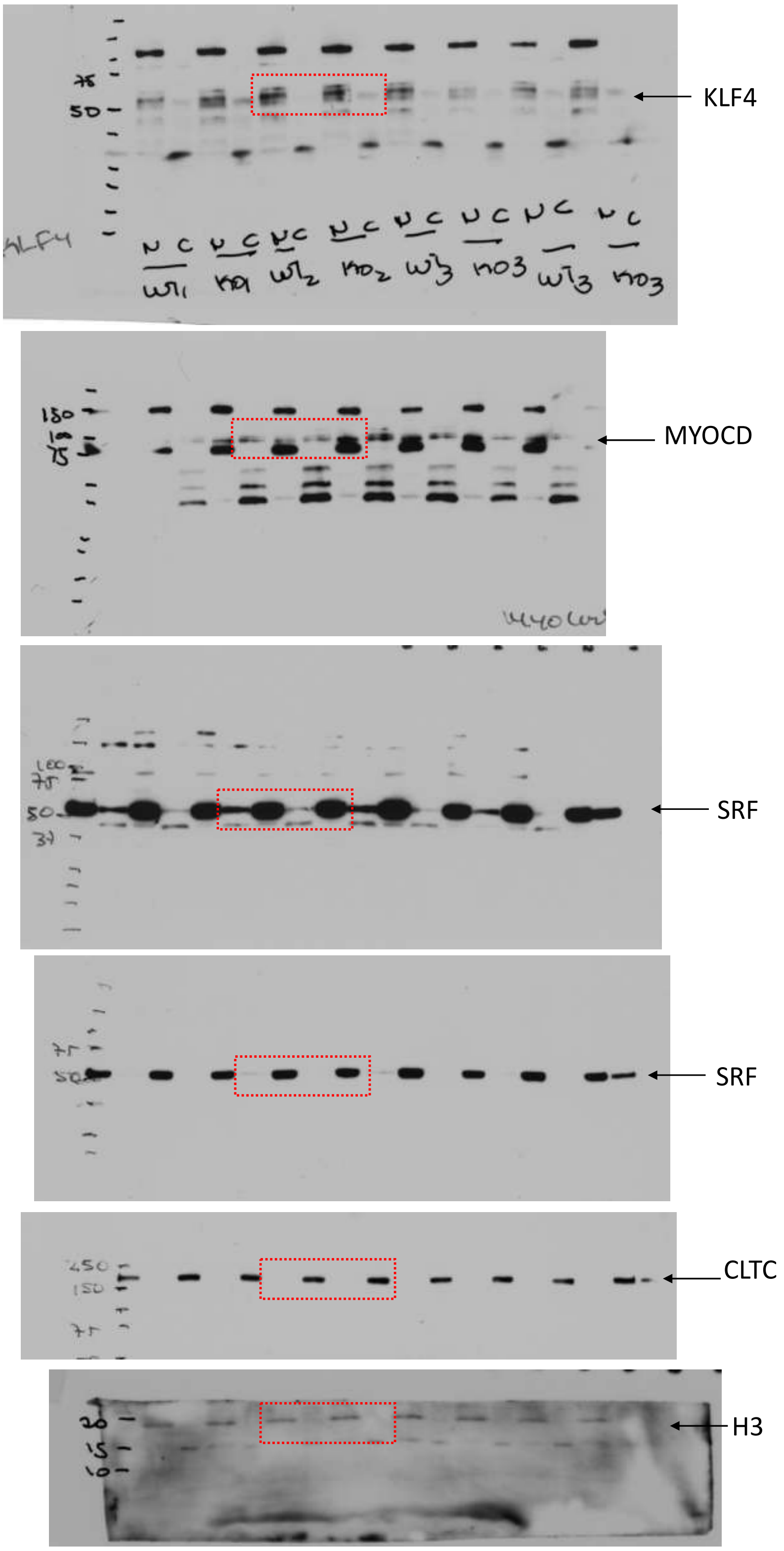

Figure 3D

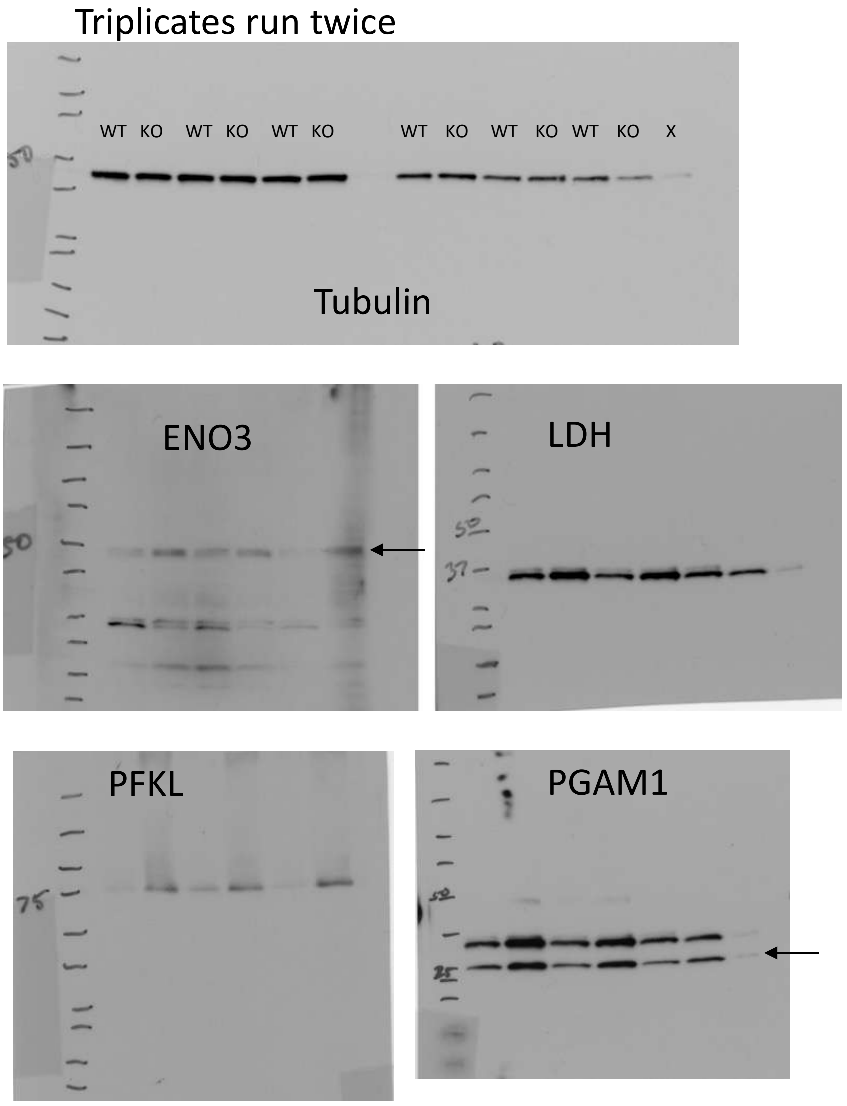

Triplicates run twice
WT KO WT KO WT KO WT KO WT KO WT KO X
50
Tubulin
ENO3
50
LDH
50
37
PFKL
75
PGAM1
50
25

Figure 3G and 3L

Order of antibodies for western blot:

1) MMP2, rabbit

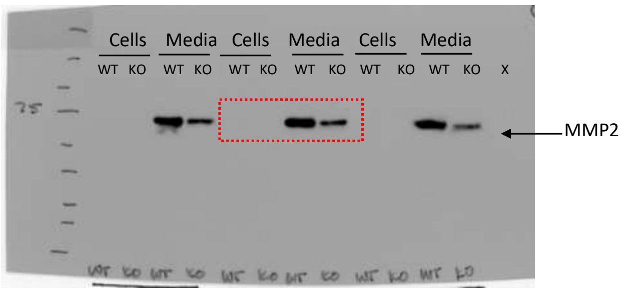

Figure 3G

Stripping

2) MMP3, rabbit

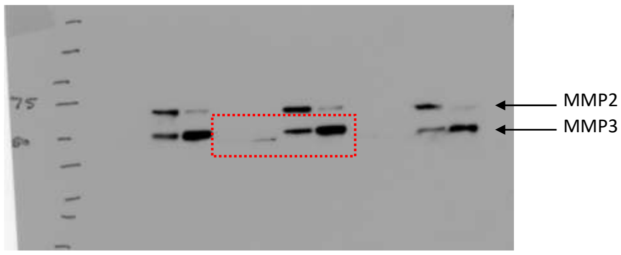

Figure 3G

Stripping

3) MMP14, rabbit

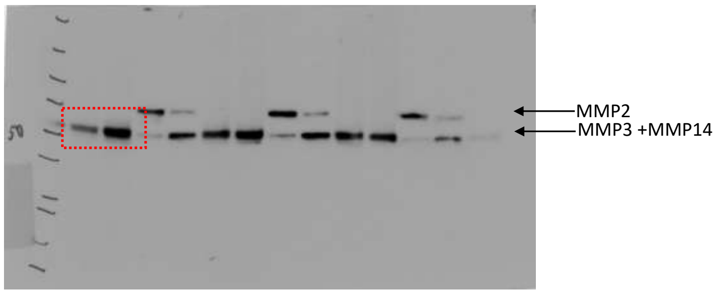

Figure 3L

Stripping

4) Cathepsin D, goat

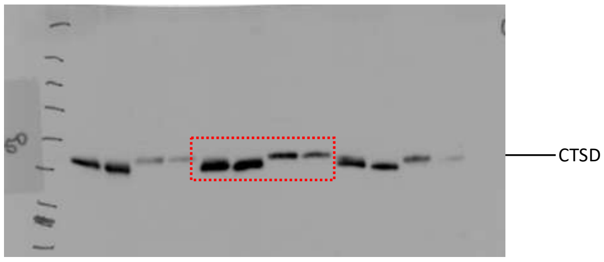

Figure 3G

5) Tubulin, mouse

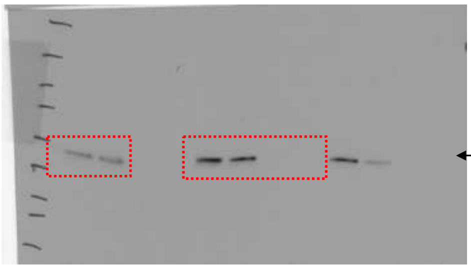

Figure 3G and 3L

6) Collagen 8, rabbit

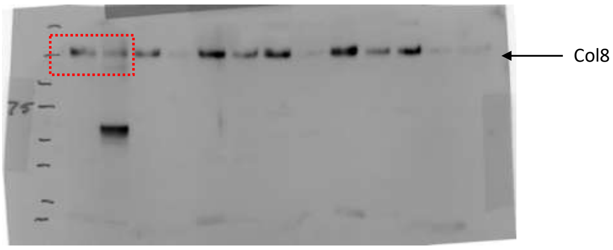

Figure 3L

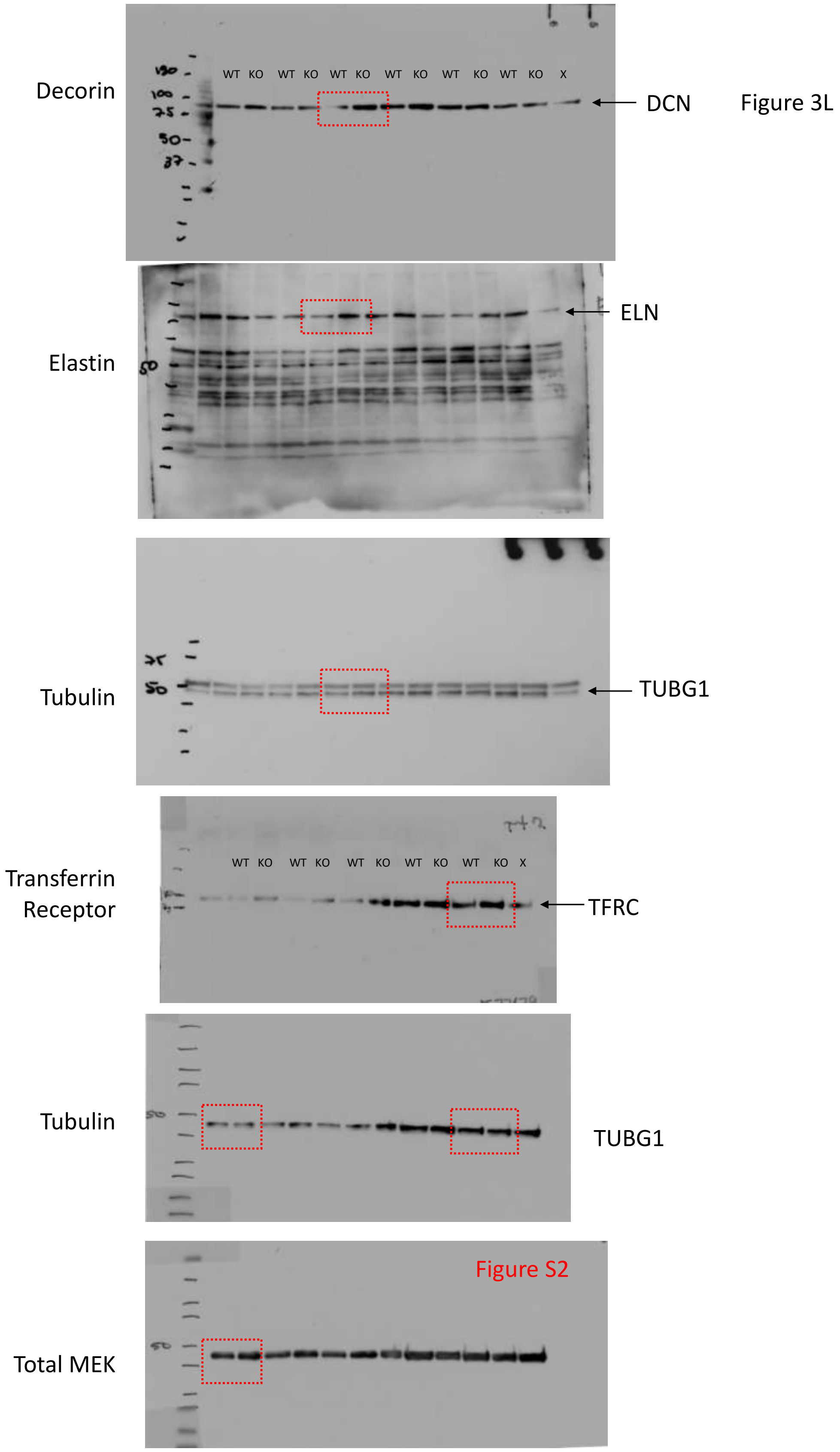

Decorin
WT KO WT KO WT KO WT KO WT KO WT KO X
DCN
Figure 3L
Elastin
ELN
Tubulin
TUBG1
Transferrin Receptor
WT KO WT KO WT KO WT KO WT KO X
TFRC
Tubulin
TUBG1
Total MEK
Figure S2

Figure 4

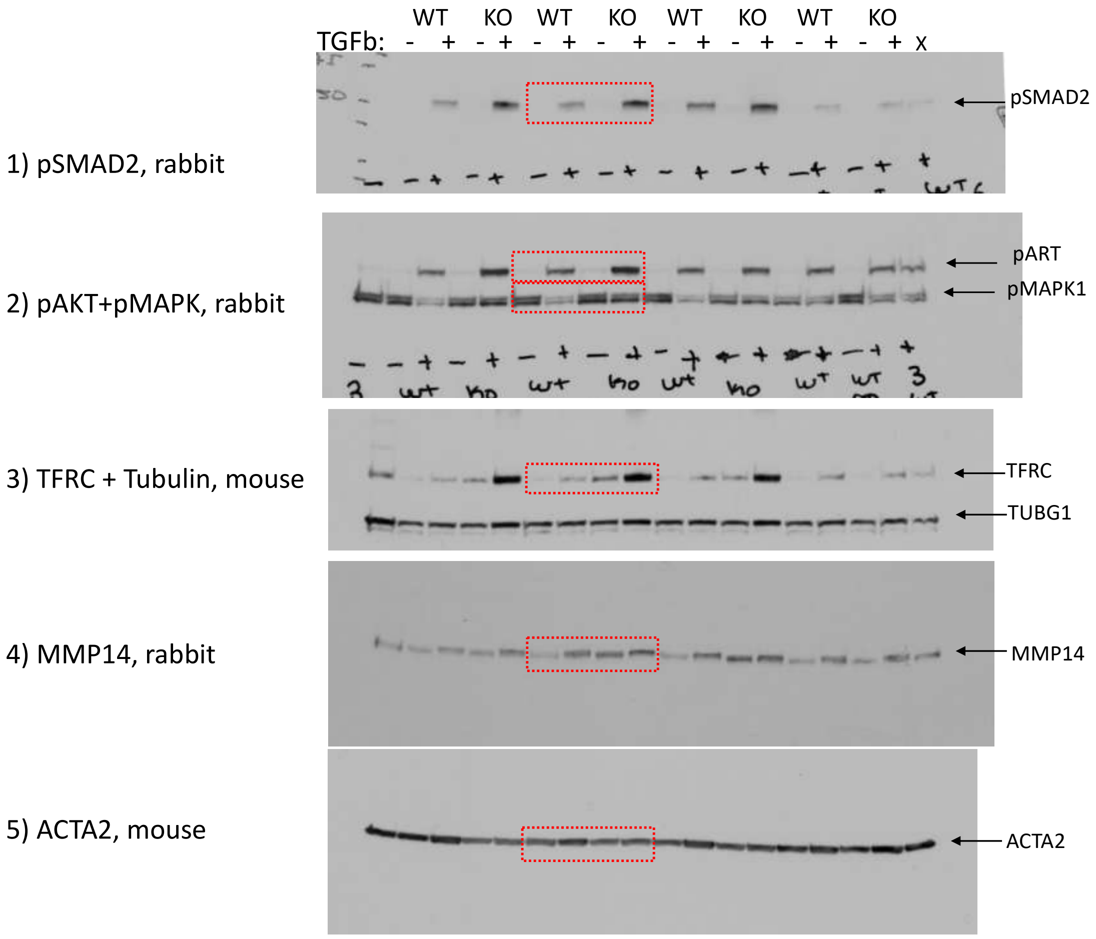

WT KO WT KO WT KO WT KO
TGFb: - + - + - + - + - + - + - + - + x
1) pSMAD2, rabbit
pSMAD2
2) pAKT+pMAPK, rabbit
pART
pMAPK1
3) TFRC + Tubulin, mouse
TFRC
TUBG1
4) MMP14, rabbit
MMP14
5) ACTA2, mouse
ACTA2

Figure 3J

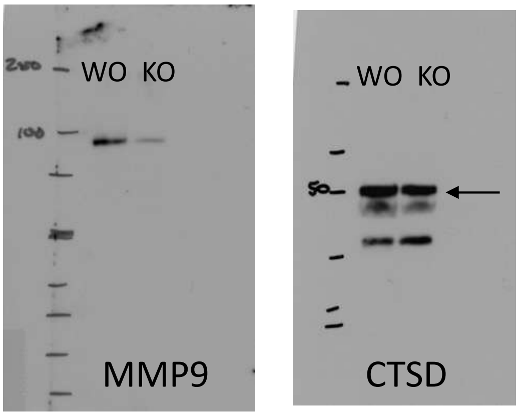

Figure 5A

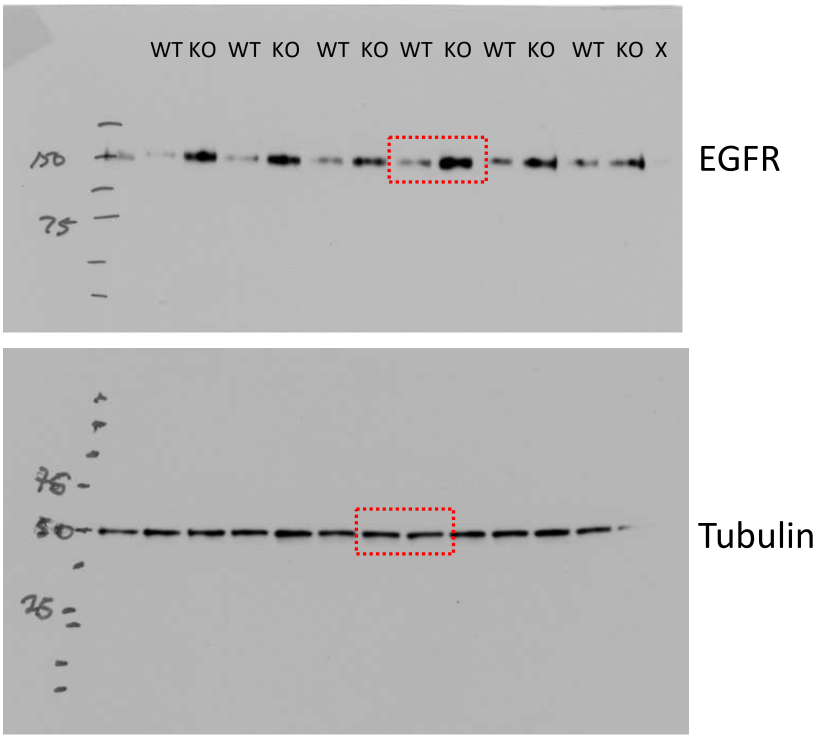

Figure 5C

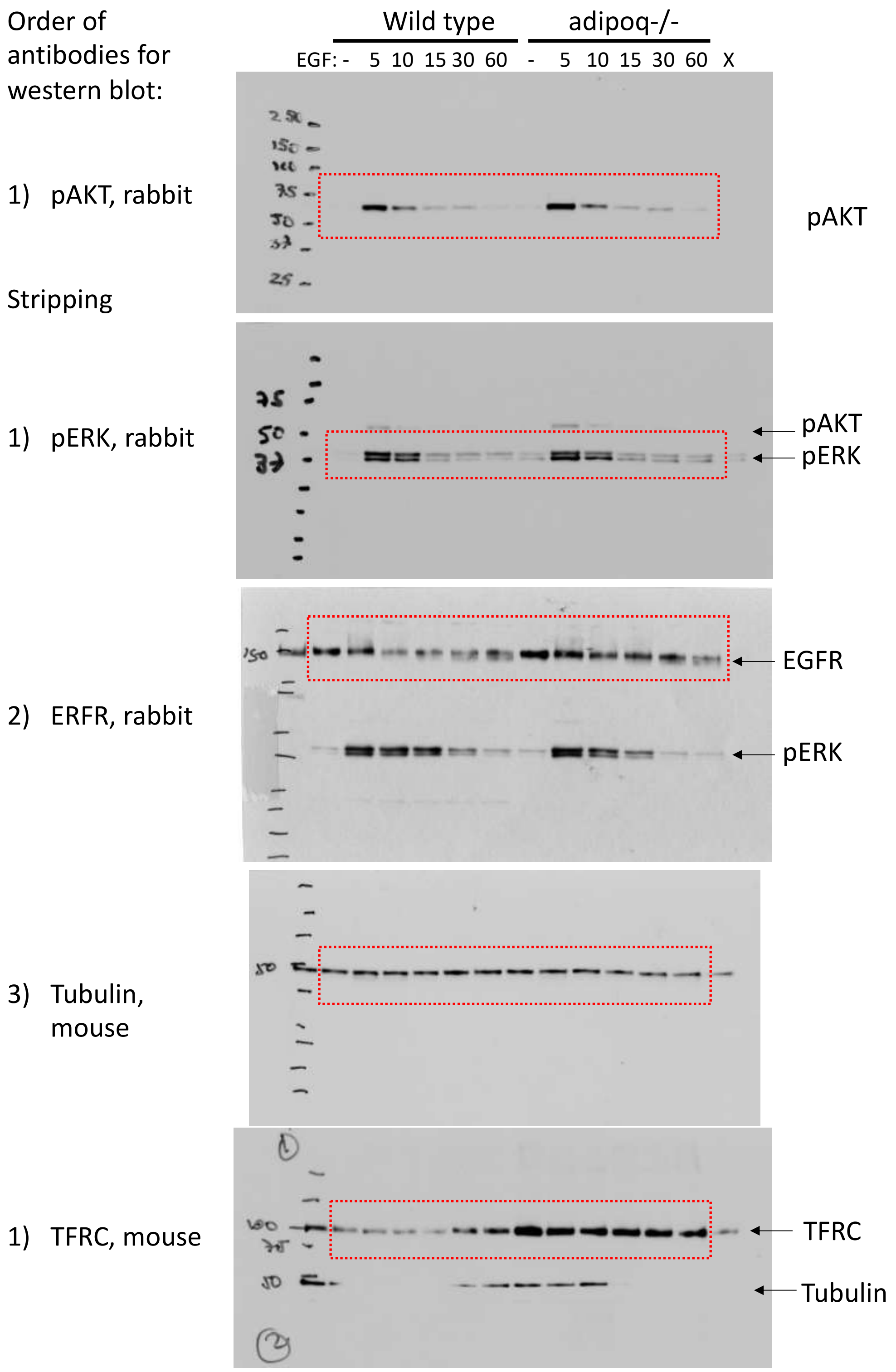

Order of antibodies for western blot:
1) pAKT, rabbit
Stripping
1) pERK, rabbit
2) ERFR, rabbit
3) Tubulin, mouse
1) TFRC, mouse
Wild type
adipoq-/-
EGF: - 5 10 15 30 60 - 5 10 15 30 60 X
pAKT
pAKT
pERK
EGFR
pERK
TFRC
Tubulin

Figure 5E

Order of antibodies for western blot:

1) EGFR, rabbit

2) Cav1, rabbit

3) Clathrin (CLTC)+ β-actin (ACTB)

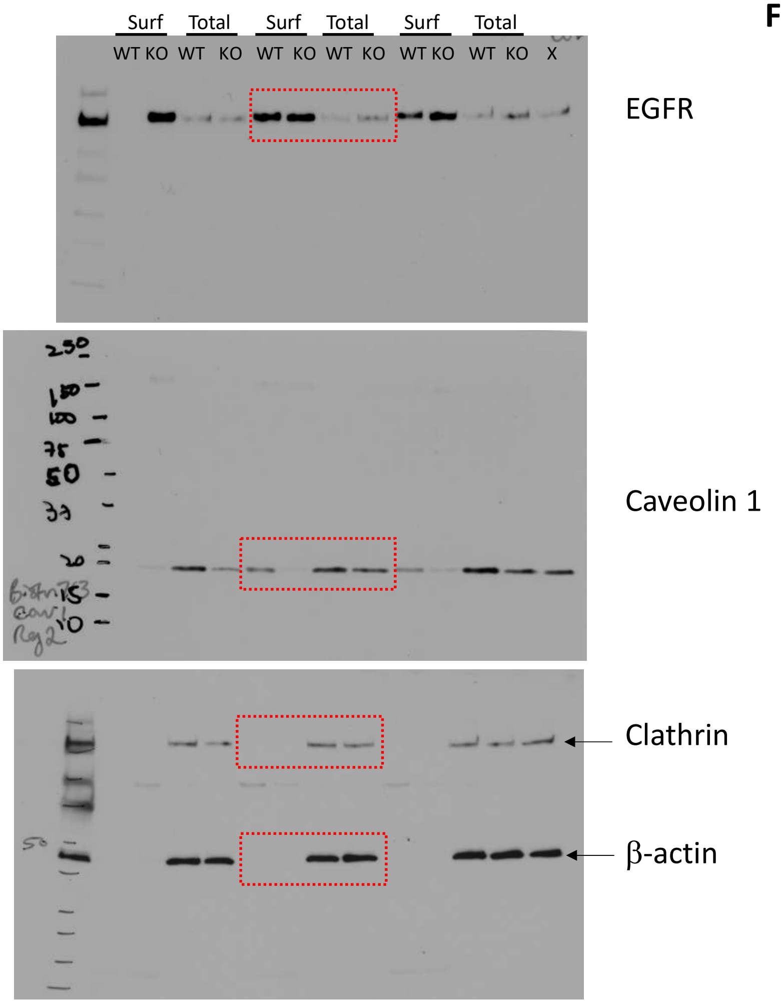

Figure 6A, mTORC1 activity

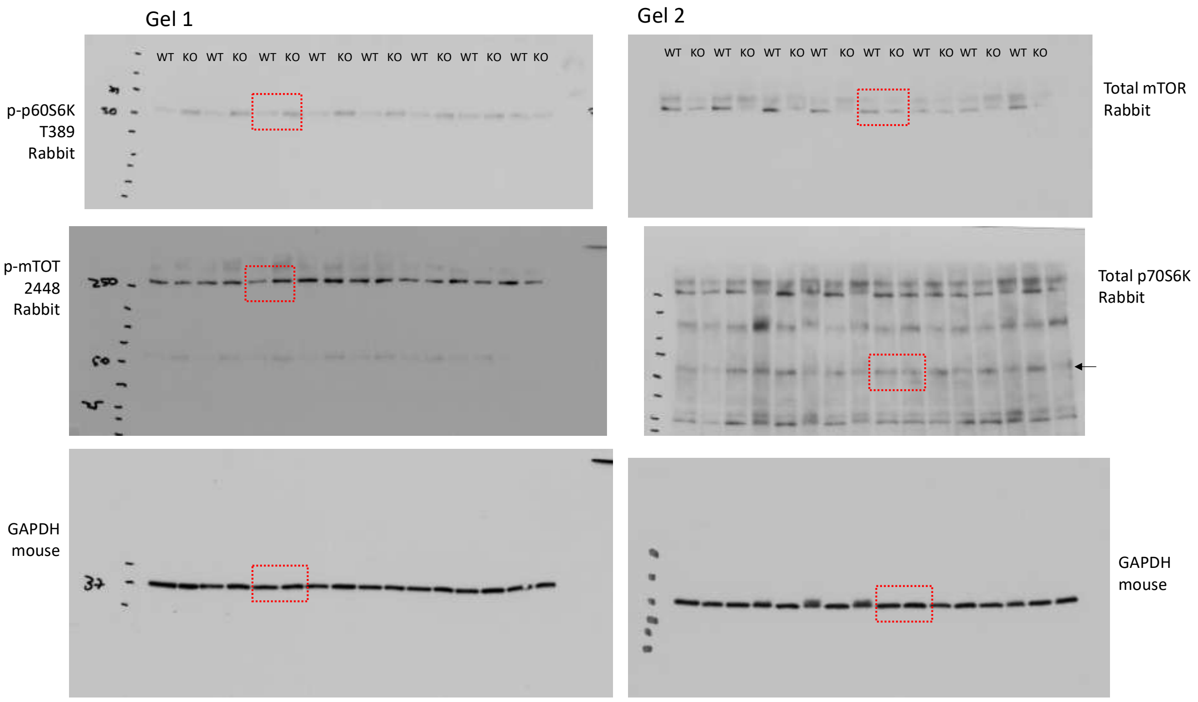

Figure 6C, Akt, MAPK1 inhibitors

Order of antibodies for western blot:

1) Tubulin, mouse

2) Cyclin D1, rabbit

3) pERK, rabbit

4) pAKT

5) SMA, mouse

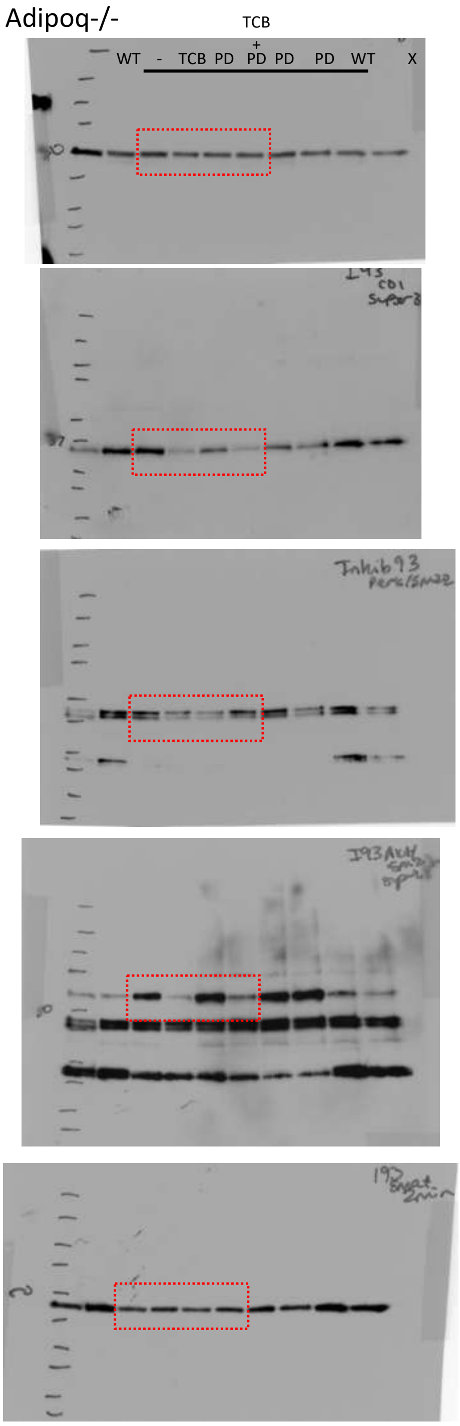

Figure 6D, EGFR, TGF-b inhibitors

Order of antibodies for western blot:

1) Cyclin D1, rabbit

1) EGFR, rabbit
SM22, rabbit

3) pAKT

4) Tubulin

5) SMA

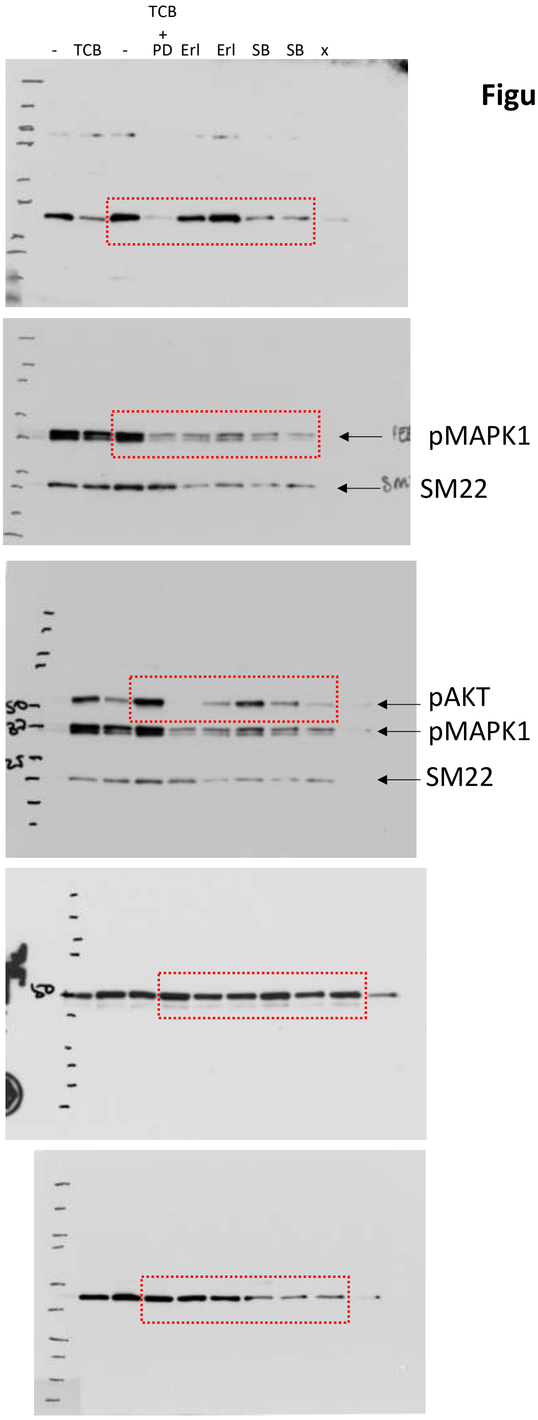

Figure 7A, Media WT and KO

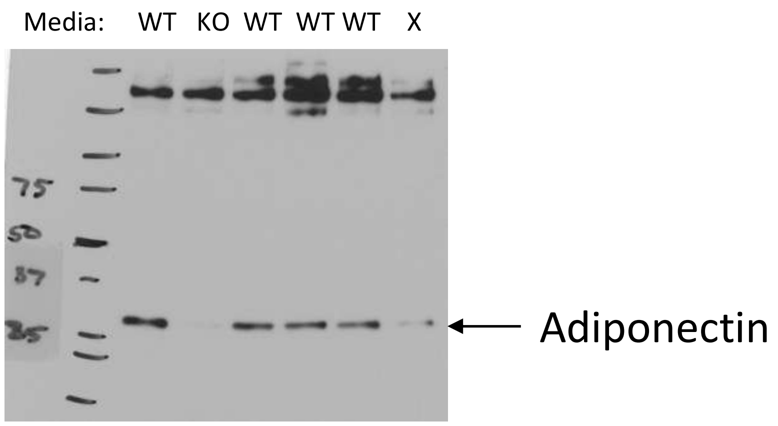

Figure 7B, Conditioned media

Order of antibodies for western blot:

1) Cyclin D1, rabbit

Stripping

2) pERK, rabbit

3) pAKT

4) Tubulin

Stripping

5) SMA

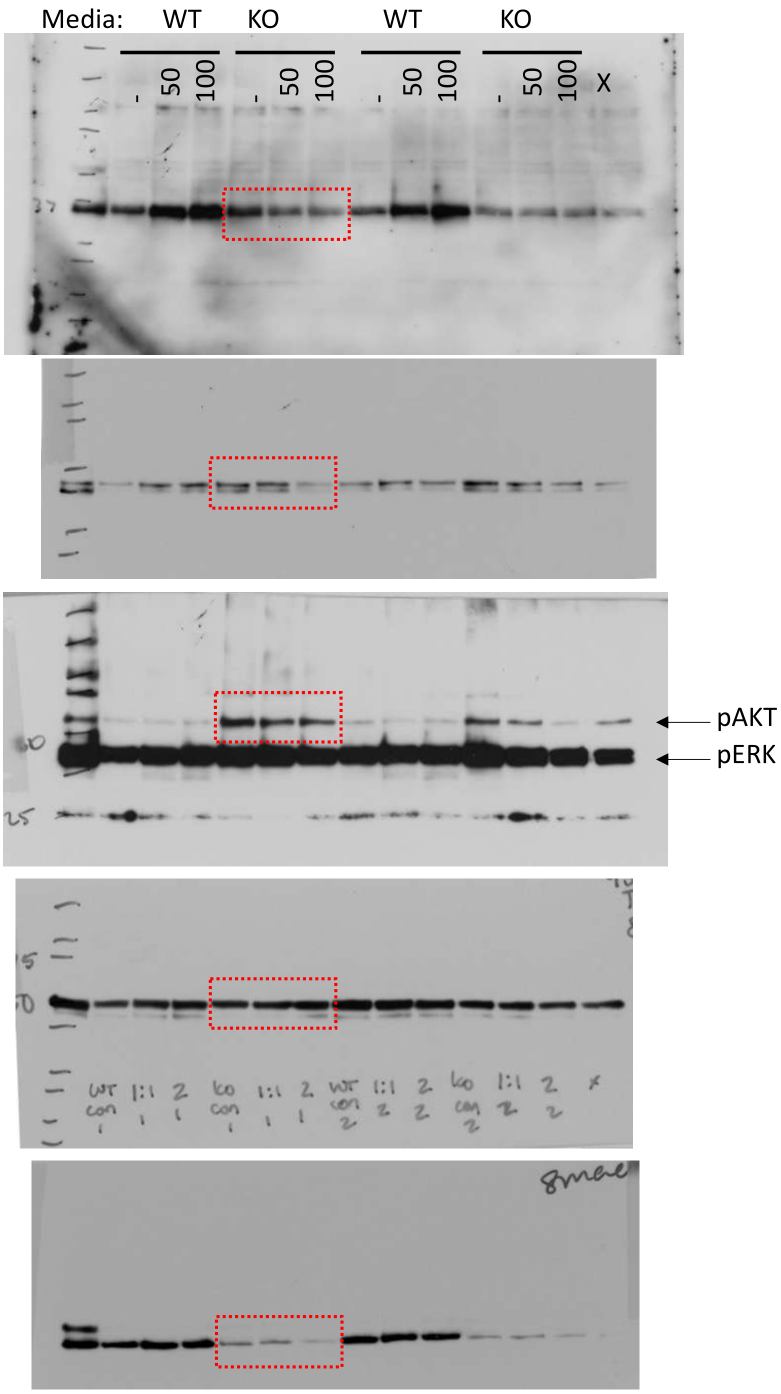

Figure 7E, AdipoRon concentration curve

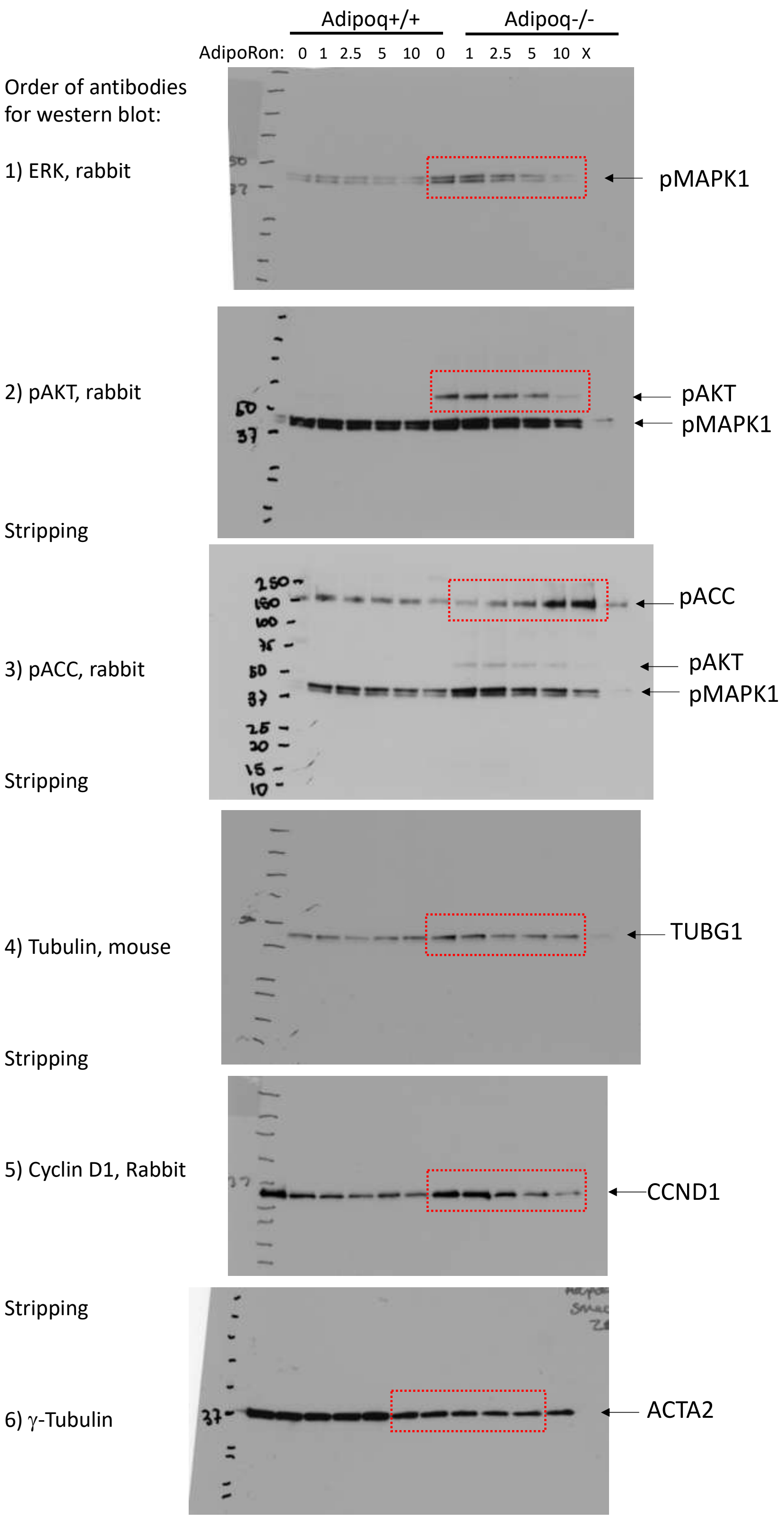

Figure 7F, metformin concentration curve

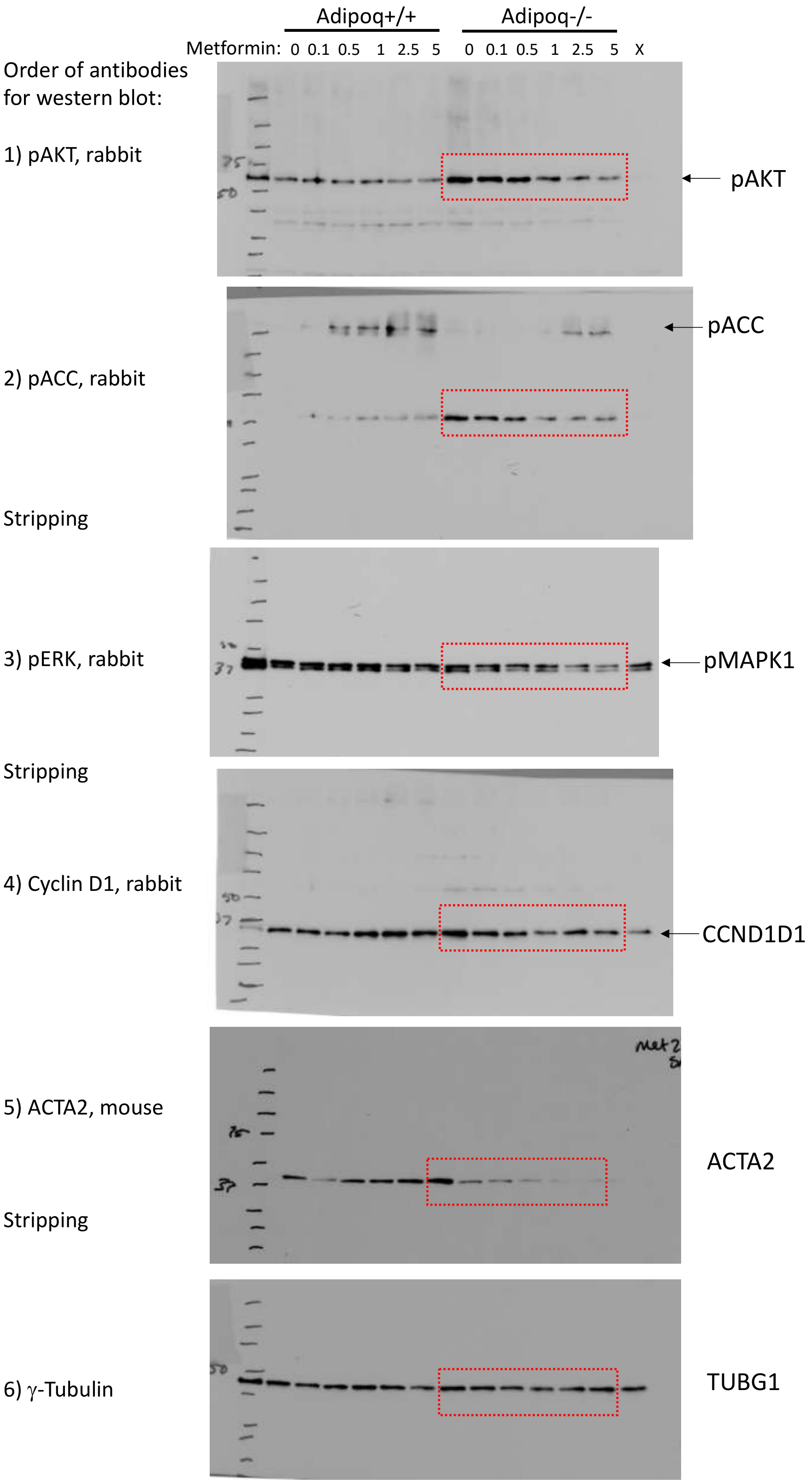

Female Aortas

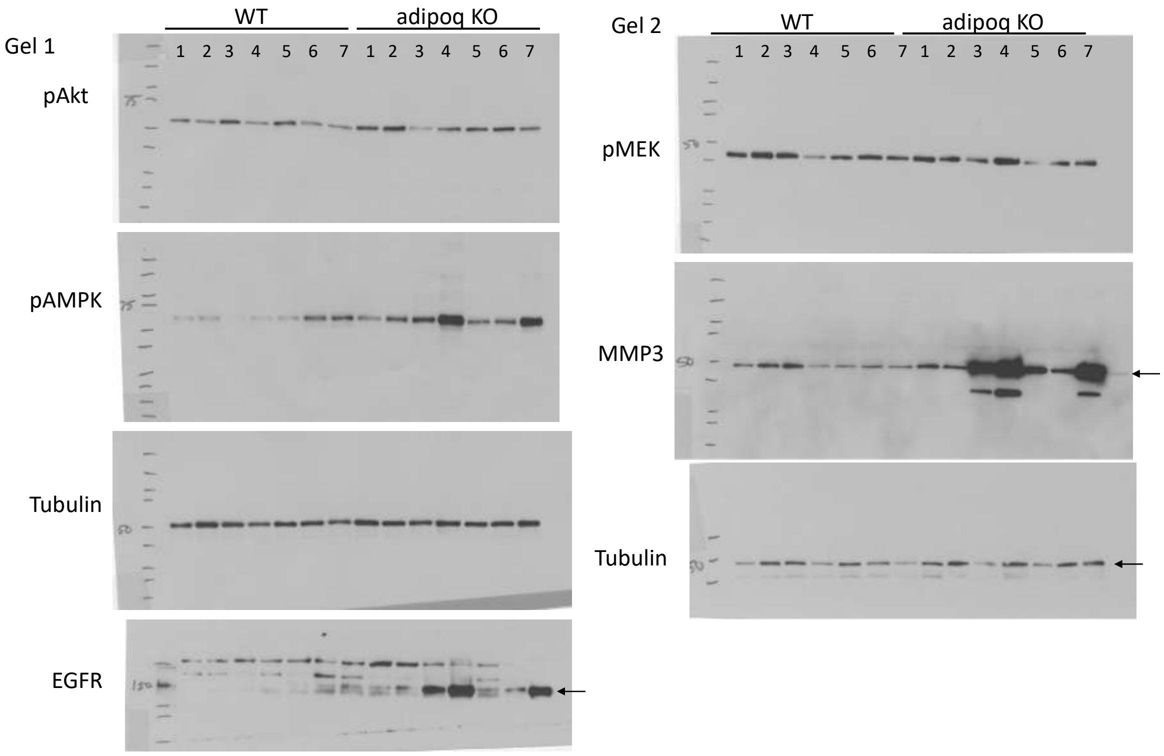

Female Aortas

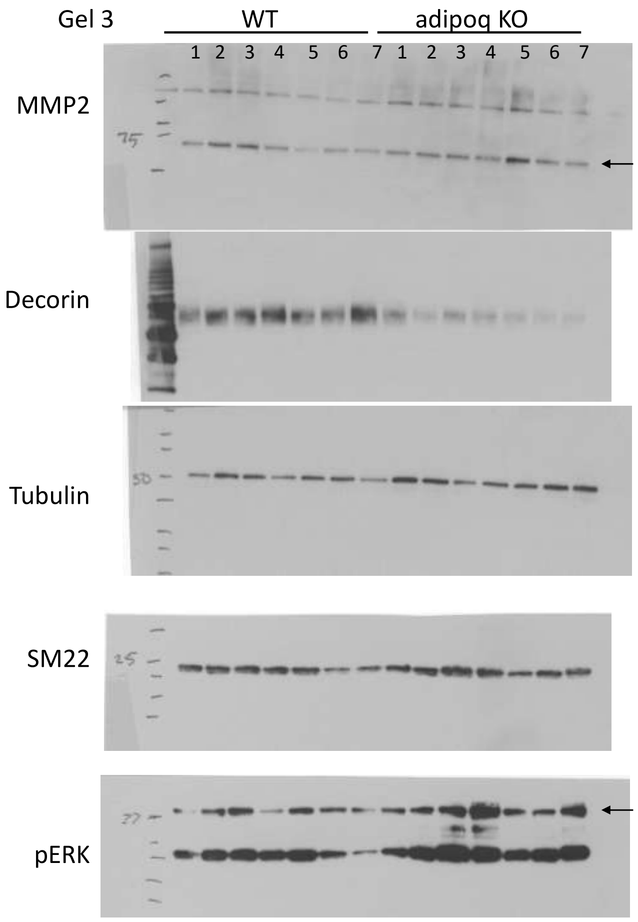

Male Aortas

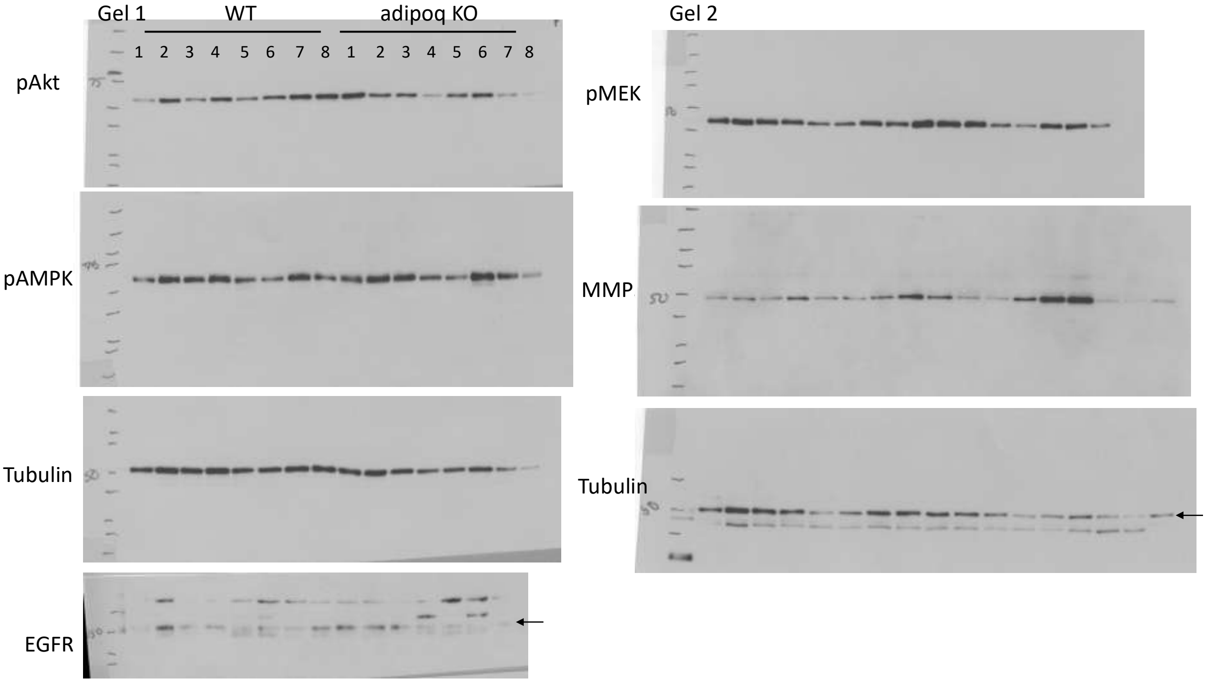
Gel 1
WT
adipoq KO
1 2 3 4 5 6 7 8 1 2 3 4 5 6 7 8
pAkt
pAMPK
Tubulin
EGFR
Gel 2
pMEK
MMP
Tubulin

Male Aortas

Gel 3

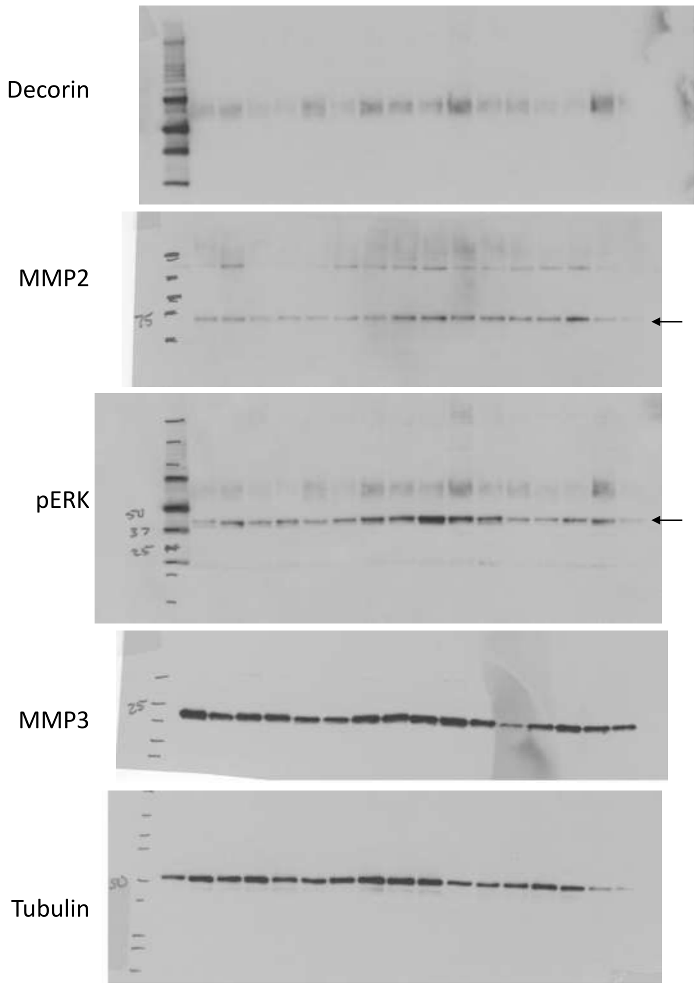

Supplement: Supplementary file 1 [file cells-12-02493-s001.zip › cells-2643754-supplementary.pdf]
